# Supplementary material for: A survey of public attitudes toward uterus transplantation, surrogacy, and adoption in Japan
Source: PLoS One. 2019 Oct 30;14(10):e0223571. doi: 10.1371/journal.pone.0223571 (PMC6821076; doi:10.1371/journal.pone.0223571)
Supplement: S1 Fig — (PPTX) [file pone.0223571.s001.pptx]

## Slide 1
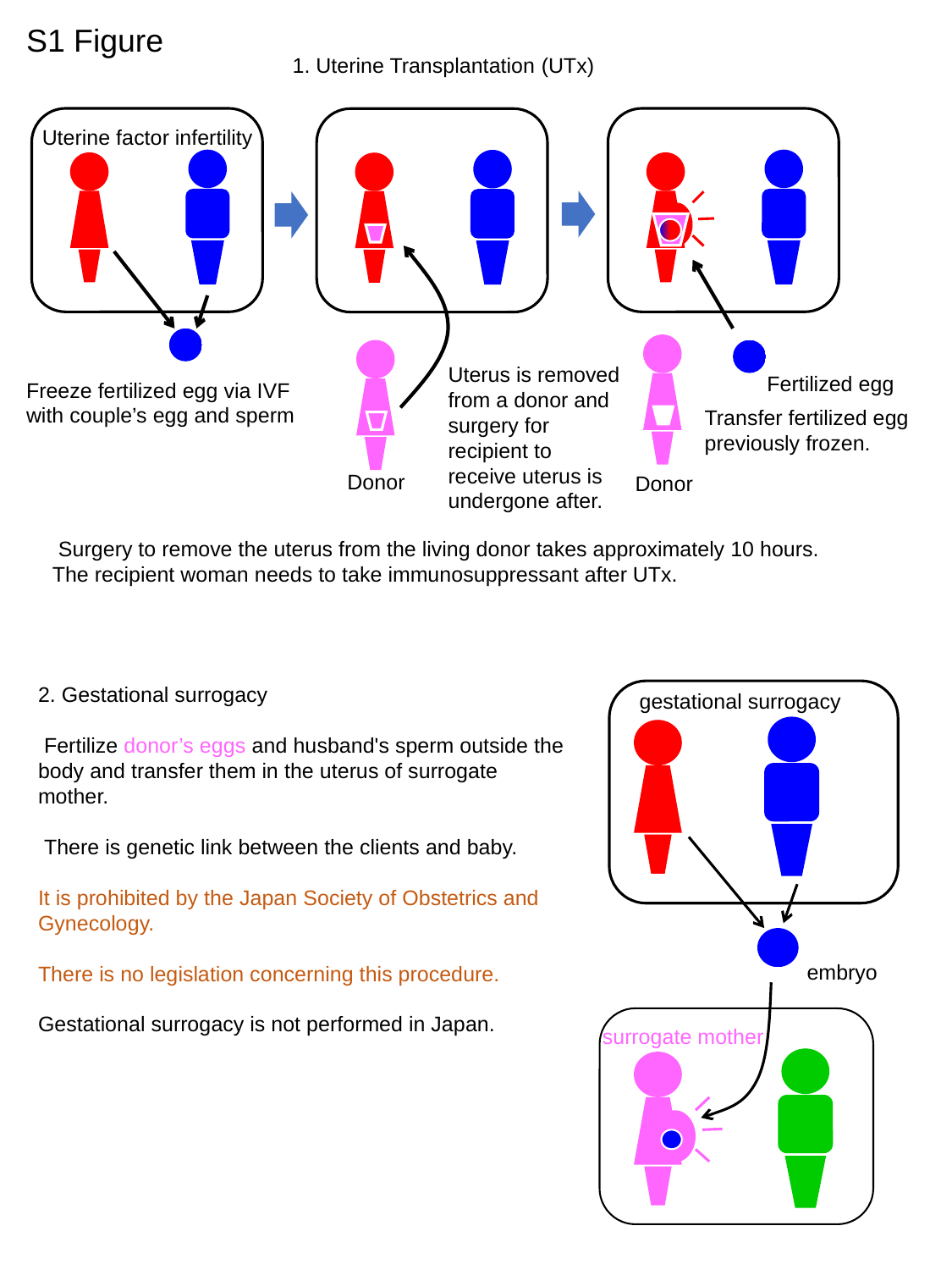

S1 Figure
1. Uterine Transplantation (UTx)
Freeze fertilized egg via IVF with couple’s egg and sperm
Uterine factor infertility
Uterus is removed from a donor and surgery for recipient to receive uterus is undergone after.
Fertilized egg
Transfer fertilized egg previously frozen.
Donor
Donor
 Surgery to remove the uterus from the living donor takes approximately 10 hours.
The recipient woman needs to take immunosuppressant after UTx.
2. Gestational surrogacy
 Fertilize donor’s eggs and husband's sperm outside the body and transfer them in the uterus of surrogate mother.
 There is genetic link between the clients and baby.
It is prohibited by the Japan Society of Obstetrics and Gynecology.
There is no legislation concerning this procedure.
Gestational surrogacy is not performed in Japan.
gestational surrogacy
embryo
surrogate mother
