# Supplementary material for: A survey of public attitudes toward uterus transplantation, surrogacy, and adoption in Japan
Source: PLoS One. 2019 Oct 30;14(10):e0223571. doi: 10.1371/journal.pone.0223571 (PMC6821076; doi:10.1371/journal.pone.0223571)
Supplement: S1 Document — (DOCX) [file pone.0223571.s002.docx]

Questionnaire

(Q.1) What is your gender? (single answer)

1. Men

2. Women

(Q.2) What is your age?

( ) years old

(Q.3) Are you married? (single answer)

*For those who lost partner or has been divorced, please answer “1. Unmarried”

1. Unmarried

2. Married

(Q.4) How many children do you have? (single answer)

1. No children

2. One

3. Two

4. Three or more.

(Q.5) Do you have any experiences suffering from infertility? (single answer)

1. I have experience

2. I have no experience

(Q.6) Have you ever received the following infertility treatment? (multiple answer)

1. I have never undergone infertility examination nor treatment

2. I have undergone infertility examination

3. Timed intercourse

4. IUI (intrauterine insemination)

5. IVF-ET (in vitro fertilization-embryo transfer)

6. ICSI (intracytoplasmic sperm infection)

(Q.7) Did you know that a woman who underwent uterine transplantation abroad got pregnant and succeeded in giving birth through cesarean section? (single answer)

1. I have never heard.

2. I have heard of it

3. I know briefly

4. I know very well

**Please read the explanation of uterine transplantation and gestational surrogacy before you answer following questions.**

①**Uterine factor infertility**

Uterine factor infertility is the general term describing infertility resulting from either abnormality of the uterus, or a complete lack of uterus. Acquired uterine disorders which happen through life also can be cause of uterine factor infertility.

② **Uterine transplantation**

Even if a woman has uterine factor infertility, assisted reproductive technology can help her to freeze fertilized egg with her own egg. Uterine transplantation is the surgical procedures whereby a healthy uterus is transplanted from a donor into a woman with absolute uterine factor infertility. After the transplantation, uterine fertility couple’s fertilized egg can be transferred in to the transplanted uterus.

Surgery to remove the uterus from the living donor is currently invasive and takes approximately 10 hours. Recipients also needs almost 4-hour surgery.

Woman who underwent uterine transplantation also have to continue taking immunosuppressant even during pregnancy and non-pregnancy period.

③ **Gestational surrogacy**

In gestational surrogacy, mature eggs are collected from wife and fertilized by husband’s sperm, and then it is transferred into the uterus of a gestational surrogate. There is a genetic link between child born and the client couple. Since the child is belonged to the surrogate mother by the law, the child is then adopted to the client couple.

The surrogate mother bears pregnancy and birth burden and risk. In Japan, the Society of Obstetrics and Gynecology does not allow gestational surrogacy so it is not actually performed in Japan. There is no legislation concerning gestational surrogacy in Japan. Some infertility couples go abroad for the gestational surrogacy.

|  | Adoption | Gestational surrogacy | Uterine transplantation (Utx) |
| --- | --- | --- | --- |
| Socially accepted or not | Allowed by law | The Japanese Society of Obstetrics and Gynecology does not allow it, but there is no legislation for gestational surrogacy in Japan. It is actually not performed in Japan | There are cases where it has been delivered overseas after UTx, but it is still in the clinical research stage. It has not been performed in Japan. |
| Parents can have genetically linked child or not. | Not possible | Possible | Possible |
| Legal family relations | Child is adopted | The mother who gave birth is, by law, the mother of the child. The client then adopts the baby. | Recipient can deliver her child and be mother by law as well. |
| Risk of surgery | None | None | Both donor and recipient need to undergo long surgeries |
| Pregnancy and delivery risk | None | Gestational surrogacy involves the medical risks of carrying a child and giving birth. | There is high risk of delivery for recipient. There is no risk for the third party. |
| Other |  | There have been cases where a surrogate mother refused to hand over a child born to a client. In addition, there was a case in which the client did not take over the baby because the born baby had a congenital disease. | It has not been performed in Japan. UTx was performed for very few cases overseas and are in clinical research stage. The safety and feasibility have not been established yet. |

Please answer the questions below, after you read the explanation above.

(Q.8) Which do you think is socially unacceptable?

1. Gestational surrogacy should not be approved.

2. UTx should not be approved

3. Neither should be approved.

4. I do not say “neither should be approved”

5. indecisive

<Respondent: person who selected 1 or 3 with Q8>

(Q.9) Why do you think that gestational surrogacy should not be approved”?

1. Because the baby born by gestational surrogacy may have a health problem.

2. Because there is a high risk for surrogate mother during pregnancy and birth.

3. Because I think that parent-child relationship will become unnatural.

4. Because there may be problems of parental authority and heritage inheritance.

5. Because pregnancy should be natural.

6. Because gestational surrogacy may be performed commercially.

7. Because women should not be used as a means of reproduction.

8. Because there are other options such as adoption.

9. Because there might be difficulty in telling the child that he/she was delivered by surrogate mother.

10. Because UTx is preferable.

11. indecisive

12. Other ( )

<Respondent : person who selected 1 or 3 with Q8>

(Q.10) Which is the first reason why you think that gestational surrogacy should not be approved”?

1. Because the baby born by gestational surrogacy may have a health problem.

2. Because there is a high risk for surrogate mother during pregnancy and birth.

3. Because I think that parent-child relationship will become unnatural.

4. Because there may be problems of parental authority and heritage inheritance.

5. Because pregnancy should be natural.

6. Because gestational surrogacy may be performed commercially.

7. Because women should not be used as a means of reproduction.

8. Because there are other options such as adoption.

9. Because there might be difficulty in telling the child that he/she was delivered by surrogate mother.

10. Because UTx is preferable.

11. indecisive

12. Other ( )

<Respondent : person who selected 2 or 3 with Q8>

(Q.11) Why do you think that UTx should not be approved?

1. UTx is not appropriate because the uterus is not a vital organ.

2. Because the risk of UTx surgery is too high.

3. Because UTx does not promise pregnancy.

4. Because the recipient is at high risk during pregnancy and birth.

5. Because pregnancy should be natural.

6. Because UTx may be performed commercially

7. Because the uterus of third party should not be used as a mean of reproduction.

8. Because there are other options such as adoption.

9. Because I think gestational surrogacy is preferrable.

10. indecisive

11. Other ( )

<Respondent : person who selected 2 or 3 with Q8>

(Q.12) Which is the first reason why you think that UTx should not be approved”?

1. UTx is not appropriate because the uterus is not a vital organ.

2. Because the risk of UTx surgery is too high.

3. Because UTx does not promise pregnancy.

4. Because the recipient is at high risk during pregnancy and birth.

5. Because pregnancy should be natural.

6. Because UTx may be performed commercially

7. Because the uterus of third party should not be used as a mean of reproduction.

8. Because there are other options such as adoption.

9. Because I think gestational surrogacy is preferrable.

10. indecisive

11. Other ( )

(Q.13) Which do you think is socially acceptable?

1. Only gestational surrogacy is acceptable (person who selected 2,4,5 with Q8 can choose)

2. Only UTx is acceptable (person who selected 1,4,5 with Q8 can choose)

3. Both of them are acceptable (person who selected 4,5 with Q8 can choose)

4. I do not say both are acceptable (person who selected 3 with Q8 automatically select this choice. Any other can choose)

5. No idea (All can choose)

<Respondent : person who selected 1or 3 with Q13>

(Q.14) Why do you think we should approve a surrogate pregnancy using the womb of a third party?

1. Because the client can receive a child without having difficult surgeries.

2. Because both the client and the surrogate mother agreed.

3. Because there is a possibility that a woman who removed the uterus due to disease or accident can have a child.

4. Because I think gestational surrogacy is acceptable anyway

5. Indecisive

6. Others

<Respondent : person who selected 1 or 3 with Q13>

(Q.15) Which is the first reason why we should approve a surrogate pregnancy using the womb of a third party??

1. Because the client can receive a child without having a difficult surgeries.

2. Because both the client and the surrogate mother agreed.

3. Because there is a possibility that a woman who removed the uterus due to disease or accident can have a child.

4. Because I think gestational surrogacy is acceptable anyway

5. Indecisive

6. Others

<Respondent : person who selected 2 or 3 with Q13>

(Q.16) Why do you think that we should approve UTx?

1. Because there is a genetic linkage with the born child and the recepient can be a birth parent.

2. The risk of pregnancy and parturition is the risk to the recipient, and does not extend to third parties such as surrogate mothers.

3. The transplanted uterus can be removed and immunosuppressant can be stopped after childbirth.

4. Because UTx will be hope for people with absolute uterine infertility.

5. Indecisive

6. Others

5. Indecisive

6. Others

<Respondent : person who selected 2 or 3 with Q13>

(Q.17) Which is the first reason why we should approve UTx?

1. Because there is a genetic linkage with the born child and the recepient can be a birth parent.

2. The risk of pregnancy and parturition is the risk to the recipient, and does not extend to third parties such as surrogate mothers.

3. The transplanted uterus can be removed and immunosuppressant can be stopped after childbirth.

4. Because UTx will be hope for people with absolute uterine infertility.

5. Indecisive

6. Others

(Q.18) If UTx is recognized socially, who is eligible to be a recipient of UTx? (multiple answer)

1. Woman who does not have vagina and uterus by nature (ex. Rokitansky syndrome)

2. Woman who lost her uterus due to cancer such disease as uterine cancer

3. Woman who lost her uterus due to benign disease such as uterine fibroid or adenomyosis

4. Woman who lost her uterine function due to endometritis or abortion surgery (ex. Usherman’s syndrome)

5. Man who wish to be pregnant

6. Indecisive

7. Other ( )

(Q.19) If UTx is recognized socially, who is eligible to be a donor of UTx? (multiple answer)

1. Recipient’s mother

2. Recipient’s sister

3. Relatives expect mother and sisters

4. Recipient’s friend

5. Third person except relatives and friend

6. Woman who has gender identity disorders

7. Brain death or heart death woman

8. Others ( )

(Q.20) If uterine transplantation is recognized socially, who is eligible to be a donor of UTx? Please select the best donor you think.

1. Recipient’s mother

2. Recipient’s sister

3. Relatives expect mother and sisters

4. Recipient’s friend

5. Third person except relatives and friend

6. Woman who has gender identity disorders

7. Brain death or heart death woman

8. Others ( )

Please answer the questions below assuming that you have spouse

Please answer the questions below assuming that your couple are suffering from absolute uterine factor infertility.

(Q.21) Do you wish for UTx if your couple are suffering from absolute uterine factor infertility?

1. Yes

2. Yes if my spouse wish for uterine transplantation

3. No even if my spouse wish for uterine transplantation

4. Indecisive

(Q.22) Do you wish for gestational surrogacy if your couple are suffering from absolute uterine factor infertility?

1. Yes

2. Yes if my spouse wish for gestational surrogacy

3. No even if my spouse wish for gestational surrogacy

4. Indecisive

(Q.23) Do you wish for child adoption if your couple are suffering from absolute uterine factor infertility?

1. Yes

2. Yes if my spouse wish for child adoption

3. No even if my spouse wish for child adoption

4. Indecisive

(Q.24) Which do you wish for the first?

1. Child adoption

2. Gestational surrogacy

3. Uterine transplantation

4. I do not wish for any choice.

5. Indecisive

Please answer the questions below assuming that you have spouse, and you have your own daughter.

<Respondent : Women only >

(Q.25) Do you want to be a donor for your daughter with absolute uterine factor infertility.?

1. Yes I wish to be a donor for our daughter.

2. Yes, I want to be a donor if there are no other donor.

3. Uterine transplantation is acceptable, but I do not want to be a donor.

4. Do not want our daughter to undergo UTx, no matter who the donor is.

5. Indecisive

<Respondent : Men only >

Do you ask your spouse to be a donor for your daughter with absolute uterine factor?

1. Yes, I want to ask my spouse to be a donor for our daughter.
2. Yes, I want to ask my spouse to be a donor if there are no other donors
3. UTx is acceptable, but I do not want my spouse to be a donor.
4. Do not want our daughter to undergo UTx, no matter who the donor is.
5. Indecisive
