# Supplementary material for: A survey of public attitudes toward uterus transplantation, surrogacy, and adoption in Japan
Source: PLoS One. 2019 Oct 30;14(10):e0223571. doi: 10.1371/journal.pone.0223571 (PMC6821076; doi:10.1371/journal.pone.0223571)
Supplement: Attachment — Submitted filename: 20190914revise letter.docx [file pone.0223571.s011.docx]

Sep 19, 2019

Editorial Board

*PLOS ONE*

Dear Editor

　We really appreciated editors and reviewers for the important suggestions, which improved our manuscript. We are also very pleased that editors and reviewers acknowledged the issue on Japanese attitudes towards uterus transplantation, gestational surrogacy, and adoption. We believe that these survey data will help to reach a national consensus on that.

Please find the revised version of our manuscript entitled “**A survey of public attitudes towards uterus transplantation, surrogacy, and adoption in Japan**.” In the attached manuscript, we described the changes made in response to the reviewer’s comments point-by-point as follows.

**Reviewer #1**

We appreciated the reviewer for the comments.

Comment #1

UTx should be changed to Surrogacy in line 309.

Response

We thank the reviewer. This was typing error, which may confuse the readers.

We corrected it as follows.

“However, some authors are skeptical of the assumption that UTx is morally superior to gestational surrogacy.”

**Reviewer #2**

We appreciated reviewers for the critical comments and important suggestions that have helped us to improve our manuscript.

Comment #1

The manuscript should be revised by a native English speaker.

Response

Thank you for your suggestion. Our manuscript was subjected to English editing company, and was revised by them. All occurrences of “towards” have been changed to “toward” for consistency.

Comment #2

The authors should describe how to isolate 200 samples from each group.

Response

We appreciated the reviewer for the important comment. Our explanation may have been confusing. We asked the internet research company for the collection of samples. The upper limit of each sample collection was determined to be 250 samples. They closed the collection, when the number of samples reached 250 samples in each group. Among them, 200 samples were selected randomly. Through this process, the internet research company extracted 1600 samples with equal segment of age and gender. After that, we received the results of extracted 1600 samples, and subsequently used them for the analysis.

We change “planned to collect….” to “asked the internet research company to collect ….” in line 93, as follows.

‘We asked the internet research company to collect 200 samples for each group of men and women in their 20's, 30’s, 40’s, and 50's.’

Comment #3

The authors should describe the definition of “infertility”.

Response

We appreciated reviewers for the important comment. In Japan, “infertility” is defined as unsuccessful attempted conception for 12 months or longer. We added “In Japan, infertility is defined as unsuccessful attempted conception for 12 months or longer.” in line 87.

Comment #4

Was the definition of “experience of infertility” confirmed by the subsequent evaluation of the reported infertility treatment?

Response

We appreciated reviewers for the comments. In this study, we asked whether respondents have any experience of infertility. The subjects were those who suffered from infertility even if they had not undergone fertility treatment or screening. In this study, of the 307 respondents with experience of infertility, 93 did not undergo infertility screening or treatment. We appreciated reviewers for the important comments. We added it in the result section in line 114, as follows.

“In this study, we asked whether respondents have any experience of infertility. The subjects were those who had infertility even if they had not undergone fertility treatment or screening. In this study, of the 307 respondents with experience of infertility, 93 did not undergo infertility screening or treatments.”

Comment #5

The authors should explain the reason why the maximum age was 60 years.

Response

Because we assumed that uterus transplantation donors and recipients could be women aged 20 to under 60, this study targeted men and women between the ages of 20 and 60. We appreciated the reviewer for the important comment. We added it in line 91, as follows.

“Because we assumed that uterus transplantation donors and recipients could be women aged 20 to under 60, this study targeted men and women between the ages of 20 and 60.”

Comment #6

The authors should give the information regarding the informed consent.

Response

We appreciated the reviewer for the important suggestion. We obtained informed consent before respondents answered questionnaire. Respondents read the study description and then choose to participate or not to participate in the study. Respondents started to answer the questionnaire when they selected “Yes” to a question of whether to participate in the survey. We also explained that respondents can stop answering if they want to stop the survey halfway. We added it in line 79, as follows.

“They provided informed consent before answering the questionnaire. Respondents read the study description and then chose to participate or not to participate in the study. Respondents started to answer the questionnaire after they selected “Yes” to a question of whether to participate in the survey. We also explained that respondents can stop answering if they want to stop the questionnaire halfway.”

Comment #7

The authors should discuss how the condition of infertility may have a significant impact of psychological well-being and sexuality.

Response

We appreciated reviewers for the suggestion. We discuss about it in line 301, as follows.

“The fact that the experience of infertility affects attitudes towards UTx and surrogacy is understandable, considering that infertility has a major impact on psychological well-being and sexuality.”

**Journal Requirements**

#1 The authors should describe the participant consent.

Response

We obtained informed consent before respondents answered questionnaire. Respondents read the study description and then choose to participate or not to participate in the study. Respondents started to answer the questionnaire when they selected “Yes” to a question of whether to participate in the survey. We also explained that respondents can stop answering and withdraw all answers if they want to withdraw from our survey halfway. We added it in line 79, as follows.

“They provided informed consent before answering the questionnaire. Respondents read the study description and then chose to participate or not to participate in the study. Respondents started to answer the questionnaire after they selected “Yes” to a question of whether to participate in the survey. We also explained that respondents can stop answering and withdraw all answers if they want to withdraw from our survey halfway.”

#2 The authors should state how to obtain the informed consent, if the survey includes minors.

Response

Our study does not include minors. We added it in line 92, as follows.

“Because we assumed that uterus transplantation donors and recipients could be women aged 20 to under 60, this study targeted men and women between the ages of 20 and 60.”

#3 The authors should add the name of internet research company in the method section.

Response

We added the name of company in line 77. The name is NEO MARKETING INC.

#4 The authors should remove the funding information from “Acknowledgments”.

Response

We removed the funding statement from Acknowledgments. We would like to add funding information in Funding Statement of online submission form, as follows.

Grant from the Ministry of Education, Culture, Sports, Science and Technology

Grant from the Japan Agency for Medical Research and Development.

We hope that the revised that the revised version of our paper is now suitable for publication in *PLOS ONE* and look forward to hearing from you.

Sincerely.

Tetsuya Hirata, M.D., Ph.D.
